# Distinct subclonal tumour responses to therapy revealed by circulating cell-free DNA

**DOI:** 10.1093/annonc/mdw278

**Published:** 2016-08-08

**Authors:** G. Gremel, R. J. Lee, M. R. Girotti, A. K. Mandal, S. Valpione, G. Garner, M. Ayub, S. Wood, D. G. Rothwell, A. Fusi, A. Wallace, G. Brady, C. Dive, N. Dhomen, P. Lorigan, R. Marais

**Affiliations:** 1 Molecular Oncology Group, Cancer Research UK Manchester Institute, Manchester; 2 The University of Manchester, The Christie NHS Foundation Trust, Manchester; 3 Clinical and Experimental Pharmacology Group, Cancer Research UK Manchester Institute, Manchester; 4 Genomic Diagnostics Laboratory, Manchester Centre for Genomic Medicine, Central Manchester NHS Foundation Trust, Manchester, UK

**Keywords:** vaginal mucosal melanoma, circulating cell-free DNA, next-generation sequencing, clonal response to therapy

## Abstract

**Background:**

The application of precision medicine in oncology requires in-depth characterisation of a patient's tumours and the dynamics of their responses to treatment.

**Patients and methods:**

We used next-generation sequencing of circulating cell-free DNA (cfDNA) to monitor the response of a *KIT* p.L576P-mutant metastatic vaginal mucosal melanoma to sequential targeted, immuno- and chemotherapy.

**Results:**

Despite a *KIT* mutation, the response to imatinib was mixed. Unfortunately, tumours were not accessible for molecular analysis. To study the mechanism underlying the mixed clinical response, we carried out whole-exome sequencing and targeted longitudinal analysis of cfDNA. This revealed two tumour subclones; one with a *KIT* mutation that responded to imatinib and a second *KIT*-wild-type subclone that did not respond to imatinib. Notably, the subclones also responded differently to immunotherapy. However, both subclones responded to carboplatin/paclitaxel, and although the *KIT*-wild-type subclone progressed after chemotherapy, it responded to subsequent re-administration of paclitaxel.

**Conclusion:**

We show that cfDNA can reveal tumour evolution and subclonal responses to therapy even when biopsies are not available.

## Abstract (precis)

The application of precision medicine requires in-depth characterisation of a patient's tumours and the dynamics of their responses to treatment. We used next-generation sequencing of cfDNA to monitor therapy responses of a metastatic vaginal mucosal melanoma and show that cfDNA can be used to monitor tumour evolution and subclonal responses to therapy even when biopsies are not available.

## introduction

Mucosal melanoma is a rare disease that accounts for ∼1.1% of all melanomas and has an incidence of 2.3 per million person-years [1]. It is characterised by a low mutation burden and a prevalence of chromosomal and copy number aberrations [2, 3]. *BRAF* mutations are rare, but amplification or mutations in the receptor tyrosine kinase *KIT* occur in ∼40% of cases [3, 4]. *KIT* aberrations are also frequent in acral melanomas and cutaneous melanomas arising over chronically sun-damaged skin, prompting clinical trials of KIT inhibitors in *KIT*-mutant melanoma [4, 5]. A phase II study of 25 patients reported objective responses to the KIT inhibitor imatinib in 54% (7/13) of *KIT*-mutant patients, versus 0% in patients with *KIT* amplifications [6]. The most common sites of mucosal melanoma are sinonasal, anorectal and urogenital, and while primary vaginal melanomas are rare, possibly because melanocytes are only found in the vaginal mucosa of ∼3% of women, the outcome for these patients is particularly poor and clinical management guidelines have not yet been agreed [1, 7].

Precision medicine offers an opportunity to improve patient care, particularly in rare cancers where clinical trials are difficult due to small patient populations. The cornerstone of precision medicine is next-generation sequencing (NGS), which can provide unbiased identification of actionable mutations. These approaches are increasingly used in clinical settings, but require access to high-quality tumour samples and knowledge of the underlying genomic landscape of the tumour to be fully effective. However, due to its rarity and proclivity for metastasising to poorly accessible visceral sites, our knowledge of the genomics of vaginal mucosal melanoma is still limited. A possible way around these limitations is to analyse the DNA released by the tumour into the patient's blood. Critically, circulating cell-free DNA (cfDNA) can accurately reflect the genomic landscapes of solid tumours and has been used to follow patient responses to therapy [8, 9], but to date these studies have largely been limited to analysing mutations identified from previous sequencing campaigns, tumour biopsies, autopsy material or accessible metastatic sites [9–11], none of which are typically available in vaginal mucosal melanoma.

We describe a patient with *KIT*-mutant vaginal mucosal melanoma who received sequential targeted, immuno- and chemotherapy. Metastatic lesions were inaccessible for molecular analysis, so we carried out whole-exome sequencing (WES) and targeted longitudinal analysis of cfDNA to monitor the patient's response to therapy. We found that the patient presented two tumour subclones that responded differently to treatment. Moreover, the cfDNA analysis predicted response to therapy and progression several weeks before these were confirmed by radiological scans. Our study shows that the dynamic analysis of cfDNA can reveal tumour heterogeneity, clonal responses to treatment and tumour evolution even when metastatic lesions are inaccessible, illustrating the enormous potential of this approach in supporting precision medicine procedures.

## patients and methods

### DNA isolation

Ethical approval was granted by the Manchester Cancer Research Centre (MCRC) Biobank Access Committee (Protocol number 13RIMA01). The patient provided written informed consent. Extraction and quantification of cfDNA was carried out as described previously [12]. Germline DNA was extracted from the remaining whole blood fraction following centrifugation using QIAamp DNA Blood Mini kits (Qiagen, Valencia, CA) by the manufacturer's instructions. DNA from formalin-fixed, paraffin-embedded (FFPE) material was isolated using GeneRead DNA FFPE kits (Qiagen) by the manufacturer's instructions.

### targeted re-sequencing

A DNA fragment spanning *KIT* p.L576 (1 ng input cfDNA) or a multiplexed panel of 15 loci (2 ng input cfDNA) were PCR amplified using GeneRead DNAseq Panel PCR Kits V2 (Qiagen). Primer sequences and amplicon characteristics are summarised in supplementary Table S1, available at *Annals of Oncology* online. The multiplex PCR panel was designed using MPprimer [13], but specific primers could not be generated for *UGT2B11* p.D458H. PCR reactions were carried out in a total volume of 40 or 50 μl (for single locus or multiplex amplifications, respectively) and contained 3 or 3.75 U of GeneRead HotStarTaq DNA polymerase (Qiagen) and a final concentration of 0.5 μM of each primer. The PCR programme included 1 cycle at 95°C for 15 min, 35 or 30 cycles of 95°C for 15 s, 55°C for 30 s and 60°C for 30 s and a final elongation step at 72°C for 10 min. PCR products were gel-purified using QIAquick Gel Extraction kits (Qiagen) and 50 or 200 ng used for library preparation. Sequencing libraries were prepared using the NEBNext Ultra DNA Library Prep Kit and NEBNext Multiplex Oligos for Illumina (New England Biolabs, Ipswich, MA) according to the manufacturer's instructions and processed on an Illumina MiSeq (Illumina, San Diego, CA).

Raw reads were first processed using Cutadapt (v. 1.8.3) to clip Illumina adapters and PCR primers. Trimmomatic (v. 0.32) was used to filter out low quality reads. The quality control processed fastq were aligned to the human genome (GRCh37) using BWA (v. 0.7.7) and the GATK (v. 3.3) framework was used for realignment around InDels. Samtools (v. 0.1.19) was used to convert the final BAMs (binary form of alignment output) to pileup format. Variants identified from pileup files using VarScan (v. 2.3.6) were then annotated using Variant Effect Predictor (VEP) (v. 73).

### whole-exome sequencing

Sequencing libraries were generated from 10 or 25 ng cfDNA, or 200 ng sheared germline DNA in Accel-NGS 2S DNA Library Kits for the Illumina Platform (Swift Biosciences, Ann Arbor, MI) by the manufacturer's instructions with the following modifications. Library amplification and indexing was carried out with KAPA HiFi HotStart PCR Kits (Kapa Biosystems, Wilmington, MA) and NEBNext Index Primers for Illumina (New England Biolabs). PCR-amplified libraries were quantified by Qubit 2.0 Fluorometer (Life Technologies, Carlsbad, CA) and 333 ng of each used for whole exome capture on SureSelectXT Reagent Kits (Agilent, Santa Clara, CA) by the manufacturer's instructions. Captured libraries were amplified using KAPA HiFi HotStart PCR Kits and PE1 (5′-AATGATACGGCGACCACCGAGATCT-3′)/PE2 (5′-CAAGCAGAAGACGGCATACGAGAT-3′) primer. Libraries were processed on an Illumina NextSeq (Illumina).

Raw fastq files were processed to remove low-quality reads using Trimmomatic (v. 0.32) and the resultant fastq files aligned to the human genome (GRCh37) using BWA aligner (v. 0.7.7). Picard (v. 1.107) was used to mark PCR duplicates in the BAM files and subsequently, the GATK framework (v. 3.3) and the InDels from 1000 Genome consortia (phase I) and SNPs from dbSNP (release 38) were used to perform realignment and mapping quality score recalibration. Somatic single-nucleotide variations (SNVs) and InDels were identified by comparison to the germline DNA pileup file using VarScan software (v. 2.3.6). Finally, the mutations were annotated for genetic context using VEP (v. 73).

### copy number analysis

For WES-based copy number analysis, per base read-depth coverage was generated from final BAM files using bedtools (v. 2.20.1) and ADTEX (v. 2.0) was used to identify regions with copy number alterations by comparing tumor and normal coverage files. Copy number calls were plotted in an exome-wide composite graph using R (v. 3.1.3). For droplet digital PCR, 25 ng DNA isolated from FFPE material or reference germline DNA were subjected to *HIn*dIII/*Eco*RI (10 U each/reaction) double-restriction enzyme digestion in a total volume of 10 μl for 1 h at 37°C. After adding 90 μl of water, 8.8 μl of the reaction was combined with 11 μl ddPCR Supermix for Probes (No dUTP) (Bio-rad, Hercules, CA), 1.1 μl of *KIT* (Hs02812715_cn) or *NLGN4X* (Hs02584007_cn)-specific probe and 1.1 μl of *TERT* TaqMan Copy Number Reference Assay (all Thermo Fisher Scientific, Waltham, MA). Droplets were generated and analysed using the QX200 AutoDG Droplet Digital PCR system according to the manufacturer's instructions (Bio-rad). For cfDNA analysis, 2 ng cfDNA or reference germline DNA (sheared to ∼150 bp using an S2 series focused-ultrasonicator, Covaris, Woburn, MA) was used directly for analysis. All reactions were carried out in triplicate and repeated at least twice.

### three-dimensional tumour measurements

The volumes of all metastatic lesions were estimated based on computed tomography (CT) image measurements using the formula for the volume calculation of ellipsoid shapes *V* = 4/3 × π(*A*/2)(*B*/2)(*C*/2), where *A* and *B* were the perpendicular diameters at maximum area representation on the axial plane and *C* was calculated from axial slices by multiplying the slice thickness (3 mm) by the number of slices between the cephalic and caudal tip of the metastasis.

## results

**Figure 1.**
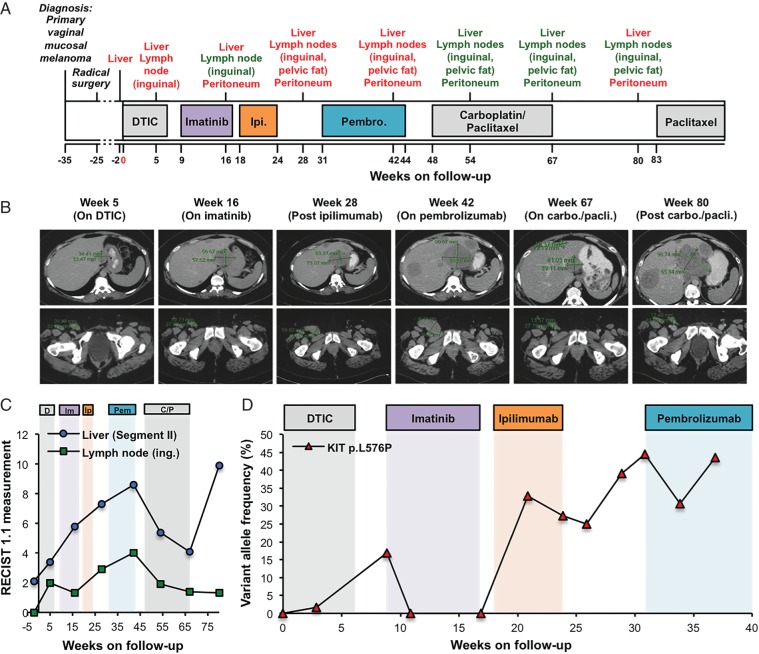
Levels of *KIT* p.L576P in the circulating cell-free DNA (cfDNA) respond to imatinib. (A) Overview of patient's treatment history, including information on initial diagnosis (35 weeks before follow-up) and surgery. Organ denominations indicate sites of metastatic disease as detected by computed tomography (CT) scans. Red font, progression; green font, response; DTIC, dacarbazine; Ipi., ipilimumab; Pembro., pembrolizumab. (B) Routine CT-generated images of the liver (top panels) and inguinal lymph node (bottom panels) at the indicated times. DTIC, dacarbazine; carbo./pacli., carboplatin/paclitaxel. (C) RECIST 1.1 measurements of a segment II liver metastasis and the inguinal (ing.) lymph node lesion during treatment with dacarbazine (D), imatinib (Im), ipilimumab (Ip), pembrolizumab (Pem) and carboplatin/paclitaxel (C/P) corresponding to (B). (D) Prospective quantification of *KIT* p.L576P VAF in cfDNA up to week 37. DTIC, dacarbazine.

The patient presented in her mid-40s with a vaginal vestibule mass, which a biopsy revealed to be a 14 mm thick, ulcerated melanoma with 29 mitoses/mm^2^. CT revealed an advanced tumour invading the rectum, so radical vaginovulvectomy, abdominoperineal resection and abdominal hysterectomy with bilateral salpingo-oophorectomy were carried out. Liver metastases developed 5 months later and following disease progression on dacarbazine (1000 mg/m^2^, two cycles), given that a *KIT* p.L576P mutation was present in the primary tumour biopsy, imatinib was administered (400 mg/day). The response to imatinib was mixed, with tumour reduction in an inguinal lymph node, but growth of a liver lesion and a new deposit appearing in the peritoneum, so imatinib was discontinued (Figure 1A–C; supplementary Figure S1, available at *Annals of Oncology* online).

Due to disease progression in the liver, the patient received ipilimumab (3 mg/kg), but discontinued after only two cycles because of disease progression and grade 3 toxicity (Figure 1A–C, supplementary Figure S1, available at *Annals of Oncology* online). The patient also progressed on pembrolizumab (2 mg/kg, five cycles) with widespread intra-abdominal disease, but she responded to paclitaxel (175 mg/m^2^) and carboplatin (AUC 6), with a 60% reduction in target lesions size (by RECIST 1.1) after two cycles and further reduction in existing lesions and no new lesions on completion of this course of treatment (week 67; Figure 1B and C). The response to paclitaxel/carboplatin was accompanied by a reduction in lactate dehydrogenase from over five times the upper limit of normal to within the normal range. However, a scan at week 80 revealed progression with growth of the tumours in the liver and peritoneum, but not the nodal sites (Figure 1A–C, supplementary Figure S1, available at *Annals of Oncology* online), so paclitaxel (175 mg/m^2^) was reinitiated and led to a clear improvement in clinical symptoms.

**Figure 2.**
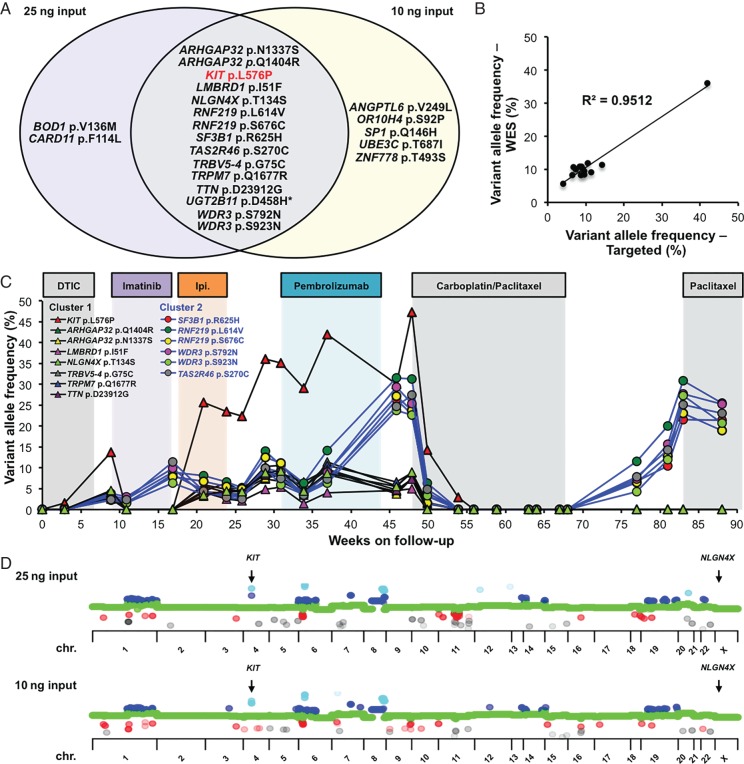
Circulating cell-free DNA (cfDNA) reveals two tumour subclones with distinct responses to therapy. (A) Venn diagram showing single-nucleotide variations identified by whole-exome sequencing (WES) of cfDNA collected at week 37. The diagram shows the mutations detected in the 25 or 10 ng input samples, with common mutations in the intersection. *KIT* p.L576P is highlighted in red to confirm its identification in both WES runs. (B) Correlation of variant allele frequencies (VAFs) detected by WES or targeted sequencing in the week 37 cfDNA sample. WES-based VAFs represent average values of the two input DNA amounts. (C) VAFs of 14 mutations (see legend within the figure) in cfDNA from samples collected at the indicated times. VAFs of mutations in clusters 1 and 2 are connected by black and blue lines, respectively, and the treatments administered are indicated above the graph. DTIC, dacarbazine; Ipi., ipilimumab. (D) Copy number variation by chromosome (chr.) based on WES from the two different input cfDNA amounts isolated at week 37 of follow-up. Grey and red dots indicate allele loss (both and one, respectively), green dots indicate normal copy number state and blue and light blue dots indicate copy number gains (three and four or more copies, respectively).

We were intrigued by the patient's mixed response to imatinib, particularly because targeted sequencing of the cfDNA revealed complete suppression of *KIT* p.L576P in the presence of imatinib and then rebounding when imatinib was withdrawn (Figure 1D). Unfortunately, as is common with vaginal mucosal melanoma, the metastatic lesions were inaccessible in this patient and only FFPE samples from the primary tumour were available for analysis, so we carried out WES of cfDNA isolated at week 37 and compared it with DNA isolated from white blood cells. We analysed 10 and 25 ng of cfDNA to provide technical replicates, and this revealed 15 reproducible somatic SNVs (Figure 2A). We developed a custom multiplexed targeted sequencing panel to monitor these SNVs in the patient's cfDNA (supplementary Table S1, available at *Annals of Oncology* online) and confirmed excellent correlation between the variant allele frequencies (VAF) determined by this panel and WES (*R*^2^ = 0.9512, Figure 2B). Note that *UGT2B11* p.D458H failed to amplify, so could not be assessed. We also observed excellent correlation between longitudinal *KIT* p.L576P analyses using the targeted single locus analysis and the multiplex panel (supplementary Figure S2, available at *Annals of Oncology* online). The results were reproducible in three independent week 37 cfDNA replicates, with a maximum VAF standard deviation of 1.62%, and germline readings not exceeding 0.27%, so a robust detection cut-off of 1% was set for subsequent analyses (supplementary Figure S3, available at *Annals of Oncology* online).

Intriguingly, longitudinal analysis of the patient's cfDNA revealed that the SNVs separated into two clusters with distinct responses to treatment (Figure 2C). Cluster 1 included eight SNVs and emerged during dacarbazine, disappeared under imatinib, returned on ipilimumab and pemprolizumab, and reduced again on carboplatin/paclitaxel (Figure 2C). Note that *KIT* p.L576P followed the same pattern as cluster 1, but at approximately fivefold higher VAF (Figure 2C). We carried out copy number analysis of the WES data and observed extensive regions of chromosomal loss and gain including focal amplification of *KIT* (Figure 2D). We confirmed the *KIT* amplification by droplet digital PCR (supplementary Figure S4, available at *Annals of Oncology* online) and reasoned that *KIT* p.L576P belonged to cluster 1, but had increased VAF due to gene amplification. Cluster 2 consisted of six SNVs, including *SF3B1* p.R625H and also emerged under dacarbazine, but in contrast to cluster 1, it increased during imatinib (Figure 2C). During ipilimumab cluster 2 decreased, but contrary to cluster 1, it increased on pembrolizumab, before falling away during carboplatin/paclitaxel (Figure 2C). Only cluster 2 SNVs re-emerged after completion of the course of carboplatin/paclitaxel, predicting relapse of this clone 3 weeks before a CT confirmed the growth of tumours in the liver and peritoneum (Figures 1B and C and 2C and supplementary Figure S1, available at *Annals of Oncology* online). Note also that cluster 2 responded to paclitaxel re-administration and the commensurate improvement in clinical symptoms.

**Figure 3.**
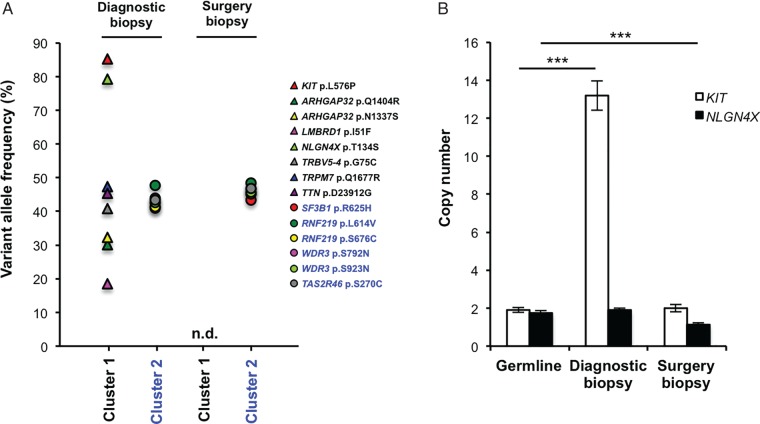
Analysis of the primary tumour reveals tumour heterogeneity. (A) Single-nucleotide variations in chromosomal DNA from the diagnostic biopsy of the primary tumour and the radical surgery 2 months after the initial biopsy. n.d., not detected. (B) Copy number determination of *KIT* and *NLGN4X* in the diagnostic biopsy and in tissue from the subsequent surgical excision when compared with germline DNA by using droplet digital PCR. ***P < 0.001.

Thus, our longitudinal analysis revealed that the patient had two distinct tumour subclones that responded differently to treatment and the patterns of tumour and cfDNA responses suggest that cluster 1 is associated with the nodal disease, whereas cluster 2 is associated with the liver and peritoneal disease. To investigate the source of subclone heterogeneity in this patient, we carried out targeted sequencing of the diagnostic biopsy that was used to identify the *KIT* p.L576P mutation. We confirmed that all 14 assessable SNVs were present in this primary tumour sample (Figure 3A), but when we examined a second FFPE specimen taken during the patient's subsequent radical surgery, we only observed cluster 2 SNVs (Figure 3A), demonstrating that the second biopsy was dominated by the cluster 2 subclone.

Consistent with the amplification of mutant *KIT* in the cluster 1 subclone, targeted sequencing revealed a high *KIT* p.L576P VAF in the clonally mixed diagnostic biopsy, but not the cluster 2-dominated surgery biopsy (Figure 3A). Unexpectedly, we also observed a high *NLGN4X* p.T134S VAF in the mixed-clone diagnostic biopsy (Figure 3A), but *NLGN4X* was not amplified during the cfDNA analysis (Figure 2D and supplementary Figure S4, available at *Annals of Oncology* online), so we carried out copy number analysis by droplet digital PCR. We confirmed the *KIT* amplification in the diagnostic biopsy, but not the surgery biopsy (Figure 3B) and while *NLGN4X* was not amplified in the diagnostic biopsy, we observed copy number loss in the surgery biopsy (Figure 3B). We conclude that the apparent high *NLGN4X* p.T134S VAF in the clonally mixed diagnostic biopsy is accounted for by the loss of a wild-type *NLGN4X* allele in the cluster 2 subclone.

## discussion

We report a case of *KIT*-mutant vaginal mucosal melanoma with a mixed response to imatinib that caused the drug to be withdrawn after only 8 weeks. Longitudinal cfDNA analysis revealed complete loss of *KIT* p.L576P under imatinib with a rapid return when imatinib was discontinued. Strikingly, this response coincided with shrinkage of a metastatic inguinal nodal lesion under imatinib and progression at this site when imatinib was discontinued, whereas the metastatic liver disease progressed through imatinib. It has been reported that cfDNA can reveal complex responses to therapy, but most previous studies have relied on targeted sequencing panels derived from biopsy or autopsy material, or knowledge of genomic landscapes of the tumours obtained from large sequencing campaigns [8, 9, 11, 14]. However, little is known about the mutational landscape of vaginal melanoma and, as is common in routine management of cancer patients, tumour biopsies from individual metastatic deposits were not available and primary tumour biopsies were preserved as FFPE specimens. Moreover, as the patient is still under treatment, autopsy biopsies were unavailable.

To overcome these challenges, we used WES of cfDNA to investigate the mechanism underlying the patient's mixed response to therapy and to search for potential therapeutic targets. Our analysis revealed that the patient presented with two tumour subclones that displayed distinct responses to targeted (imatinib) and immuno (pembrolizumab)-therapies. Importantly, targeted sequencing of the diagnostic biopsy did not reveal the presence of two subclones, and sequencing of the surgery biopsy revealed only one of the subclones. This illustrates the limitations in analysing solid tumour biopsies, as single biopsies cannot reveal subclonal mixtures in a tumour unless single-cell analysis is carried out. Moreover, single tumour biopsies may provide incomplete genetic information if there is subclonal dominance within the sampled region and, as in our case, this could result in actionable mutations being missed. Similarly, single cfDNA samples cannot reveal subclonal populations or their responses to therapy. However, the combination of WES and targeted longitudinal monitoring of the cfDNA revealed not only the presence of the individual subclones, but also their genetic constitution and distinct responses to therapy. Critically, we achieved this level of insight even though the patient's metastatic lesions were inaccessible.

Our data are consistent with the mixed response to imatinib and indicate that it was effective, albeit in a single clone. We were not able to determine the relationship between the subclones and it is curious that they appeared not to share any common mutations. One possibility is that they were related through truncal drivers such as copy number gains and losses, fusion genes or epigenetic modifications that are characteristic of mucosal melanoma, but not revealed by longitudinal targeted sequencing. Alternatively, the clones may not have been related, but we confirmed that both were present in at least part of the primary tumour, suggesting that they emerged from the same site. Critically, irrespective of their origins, we identified potential therapeutic targets for both clones in the cfDNA; imatinib was effective against the cluster 1 clone, and a mutation in *SF3B1*, a component of the spliceosome and a validated drug target [15], was discovered in the cluster 2 clone.

In conclusion, we show that WES and longitudinal, targeted analysis of cfDNA can overcome the need for biopsies to reveal subclonal responses to treatment. Despite the clinical diagnosis of disease progression, we showed that imatinib controlled one subclone, and its withdrawal allowed this subclone to re-emerge. This suggests that imatinib would be a valid option for combination therapy for this patient, although targeted options for cluster 2 are not yet in the clinic and potential compound toxicity may limit the use of unproven combination therapies in patients. Our analysis also provided an early indication that both subclones responded to chemotherapy and it predicted relapse of one of the subclones before it was revealed by a radiological scan. The key to precision medicine is a better understanding of tumour complexity and the dynamics of response to treatment and we show that this can be provided by the analyses we carried out. Moreover, the approach is convenient for the patient, simple to implement and relatively inexpensive. Although alternative treatment options were not available for our patient, we provide an important proof of principle of how in-depth cfDNA analysis can improve patient care through implementation of personalised medicine.

## funding

This work was supported by the Wellcome Trust (100282/Z/12/Z); and the Cancer Research UK Manchester Institute (C5759/A12328). SV was supported by the ESMO Clinical Research Fellowship with the aid of a grant from Novartis. Any views, opinions, findings, conclusions, or recommendations expressed in this material are those solely of the author(s) and do not necessarily reflect those of ESMO or Novartis.

## disclosure

The authors have declared no conflicts of interest.

## Supplementary Material

Supplementary Data
