# Supplementary material for: Distinct subclonal tumour responses to therapy revealed by circulating cell-free DNA
Source: Ann Oncol. 2016 Aug 8;27(10):1959–65. doi: 10.1093/annonc/mdw278 (PMC5035787; doi:10.1093/annonc/mdw278)
Supplement: Supplementary Data [file supp_27_10_1959__index.html]

Distinct sub-clonal tumour responses to therapy revealed by circulating cell-free DNA — Distinct subclonal tumour responses to therapy revealed by circulating cell-free DNA — Distinct subclonal tumour responses to therapy revealed by circulating cell-free DNA — Supplementary Data 

# Distinct subclonal tumour responses to therapy revealed by circulating cell-free DNA

## Supplementary Data

Supplementary Data

- Supplementary Data - Docx file
- Supplementary Figure\_legends - docx file
- Supplementary Figure 1 - tif file
- Supplementary Figure 2 - tif file
- Supplementary Figure 3 - tif file
- Supplementary Figure 4 - tif file
- Supplementary Table 1 - docx file
