# Supplementary material for: Distinct subclonal tumour responses to therapy revealed by circulating cell-free DNA
Source: Ann Oncol. 2016 Aug 8;27(10):1959–65. doi: 10.1093/annonc/mdw278 (PMC5035787; doi:10.1093/annonc/mdw278)
Supplement: Supplementary Data [file supp_mdw278_mdw278supp.docx]

**Supplementary Methods**

**Targeted re-sequencing data analysis**

Raw reads were first processed using Cutadapt (v. 1.8.3) to clip Illumina adapters and PCR primers. Trimmomatic (v. 0.32) was used to filter out low quality reads. The quality control processed fastq were aligned to the human genome (GRCh37) using BWA (v. 0.7.7) and the GATK (v. 3.3) framework was used for realignment around InDels. Samtools (v. 0.1.19) was used to convert the final BAMs (binary form of alignment output) to pileup format. Variants identified from pileup files using VarScan (v. 2.3.6) were then annotated using Variant Effect Predictor (VEP) (v. 73).

**Whole exome sequencing data analysis**

Raw fastq files were processed to remove low quality reads using Trimmomatic (v. 0.32) and the resultant fastq files aligned to the human genome (GRCh37) using BWA aligner (v. 0.7.7). Picard (v. 1.107) was used to mark PCR duplicates in the BAM files and subsequently, the GATK framework (v. 3.3) and the InDels from 1000 Genome consortia (phase I) and SNPs from dbSNP (release 38) were used to perform realignment and mapping quality score recalibration. Somatic SNVs and InDels were identified by comparison to germline DNA BAM files using VarScan software (v. 2.3.6). Finally the mutations were annotated for genetic context using VEP (v. 73).

**Three-dimensional tumour measurements**

The volumes of all metastatic lesions were estimated based on CT image measurements using the formula for the volume calculation of ellipsoid shapes V=4/3*π(A/2)(B/2)(C/2) where A and B were the perpendicular diameters at maximum area representation on the axial plane and C was calculated from axial slices by multiplying the slice thickness (3mm) by the number of slices between the cefalic and caudal tip of the metastasis.
