# Supplementary material for: Distinct subclonal tumour responses to therapy revealed by circulating cell-free DNA
Source: Ann Oncol. 2016 Aug 8;27(10):1959–65. doi: 10.1093/annonc/mdw278 (PMC5035787; doi:10.1093/annonc/mdw278)
Supplement: Supplementary Data [file supp_mdw278_mdw278supp_fig_legends.docx]

**Supplementary Figure S1.** Estimation of total disease volume, classified by metastatic site.

Volumes of individual metastases were estimated based on CT image measurements using the formula for the volume calculation of ellipsoid shapes and reported as the sum of all volumes per metastatic site. DTIC, dacarbazine; Ipi, ipilimumab; Pembro, pembrolizumab.

**Supplementary Figure S2.** Comparison of single locus and multiplex detection of *KIT* p.L576P VAF.

**(A)** Longitudinal analysis of *KIT* p.L576P VAF up to week 37 of follow-up based on single locus or multiplex PCR enrichment.

**(B)** Correlation of *KIT* p.L576P VAFs detected by longitudinal single locus or multiplexed targeted sequencing.

**Supplementary Figure S3.** Assay reproducibility and sensitivity.

Detection cut-off and reproducibility of the targeted sequencing approach were estimated by independent analyses of three week 37 cfDNA samples (pink squares) and of germline DNA (black dots). Error bars represent averages of three replicate values plus/minus standard deviation.

**Supplementary Figure S4.** Confirmation of *KIT* and *NLGN4X* copy number alterations in cfDNA.

Copy number determination of *KIT* and *NLGN4X* in germline DNA and week 37 cfDNA using droplet digital PCR. *** = *P<0.001*
