# Supplementary figures and images for: Distinct subclonal tumour responses to therapy revealed by circulating cell-free DNA
Source: Ann Oncol. 2016 Aug 8;27(10):1959–65. doi: 10.1093/annonc/mdw278 (PMC5035787; doi:10.1093/annonc/mdw278)

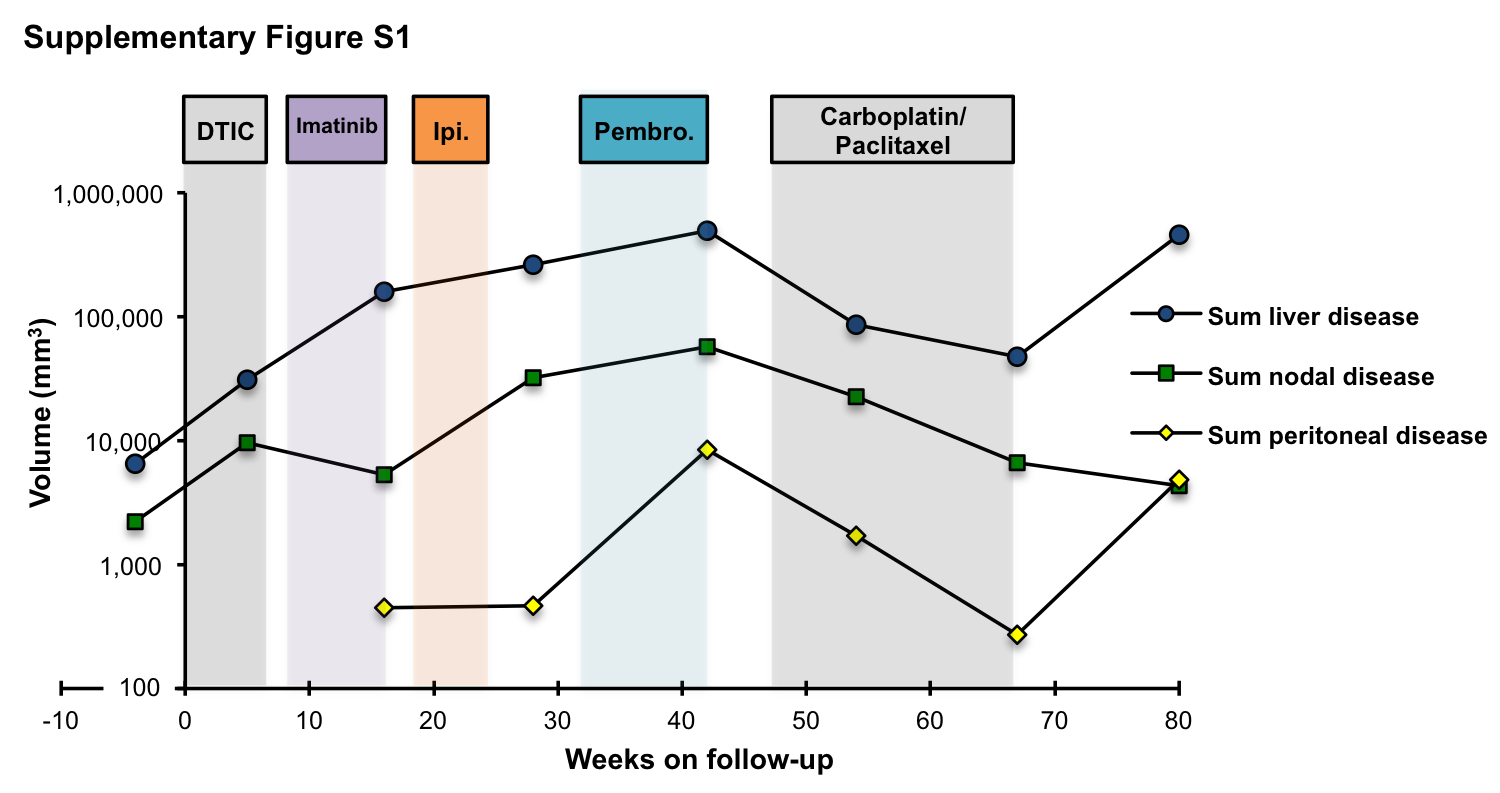

Supplement: Supplementary Data [file supp_mdw278_mdw278supp_fig1.tif]

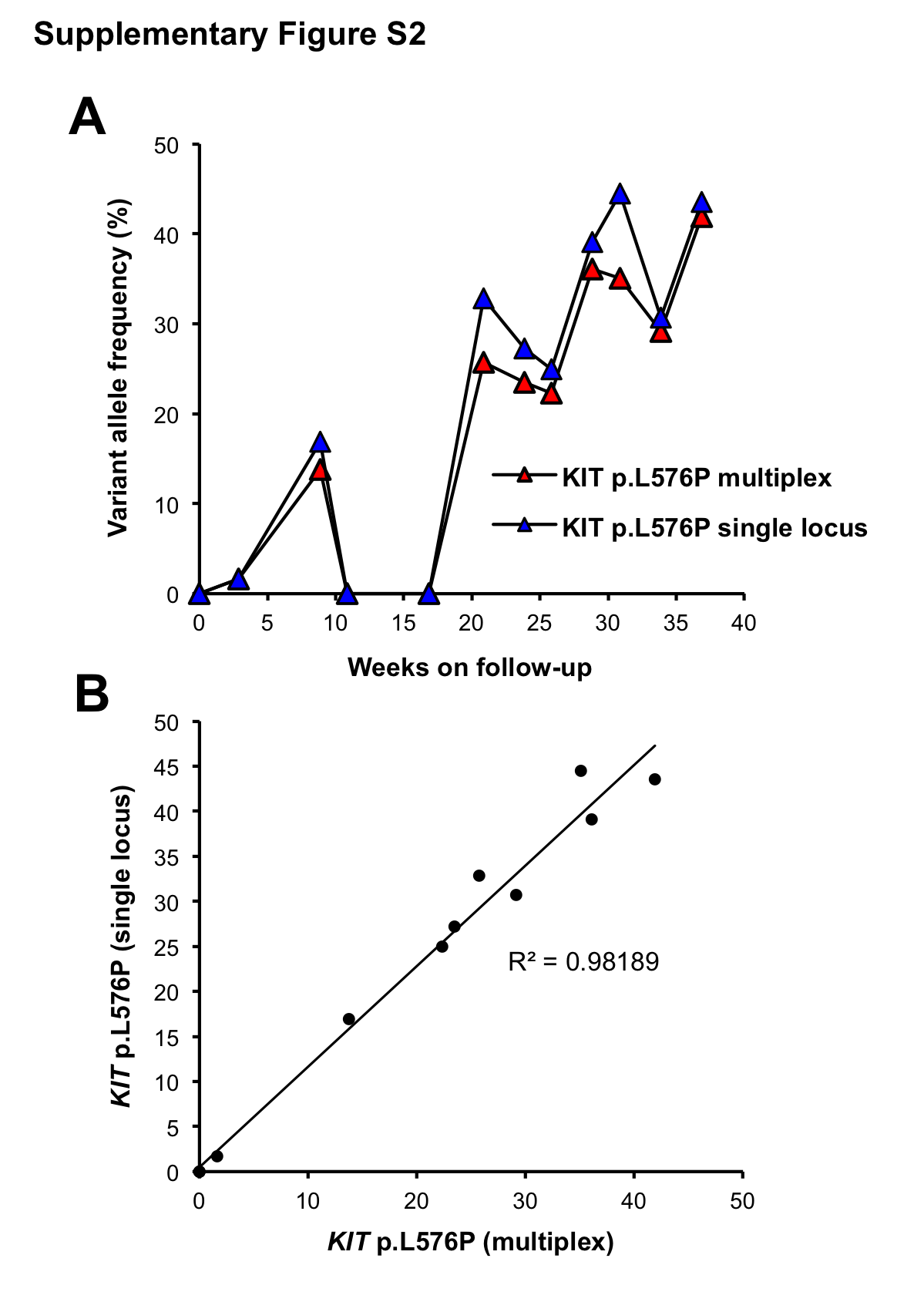

Supplement: Supplementary Data [file supp_mdw278_mdw278supp_fig2.tif]

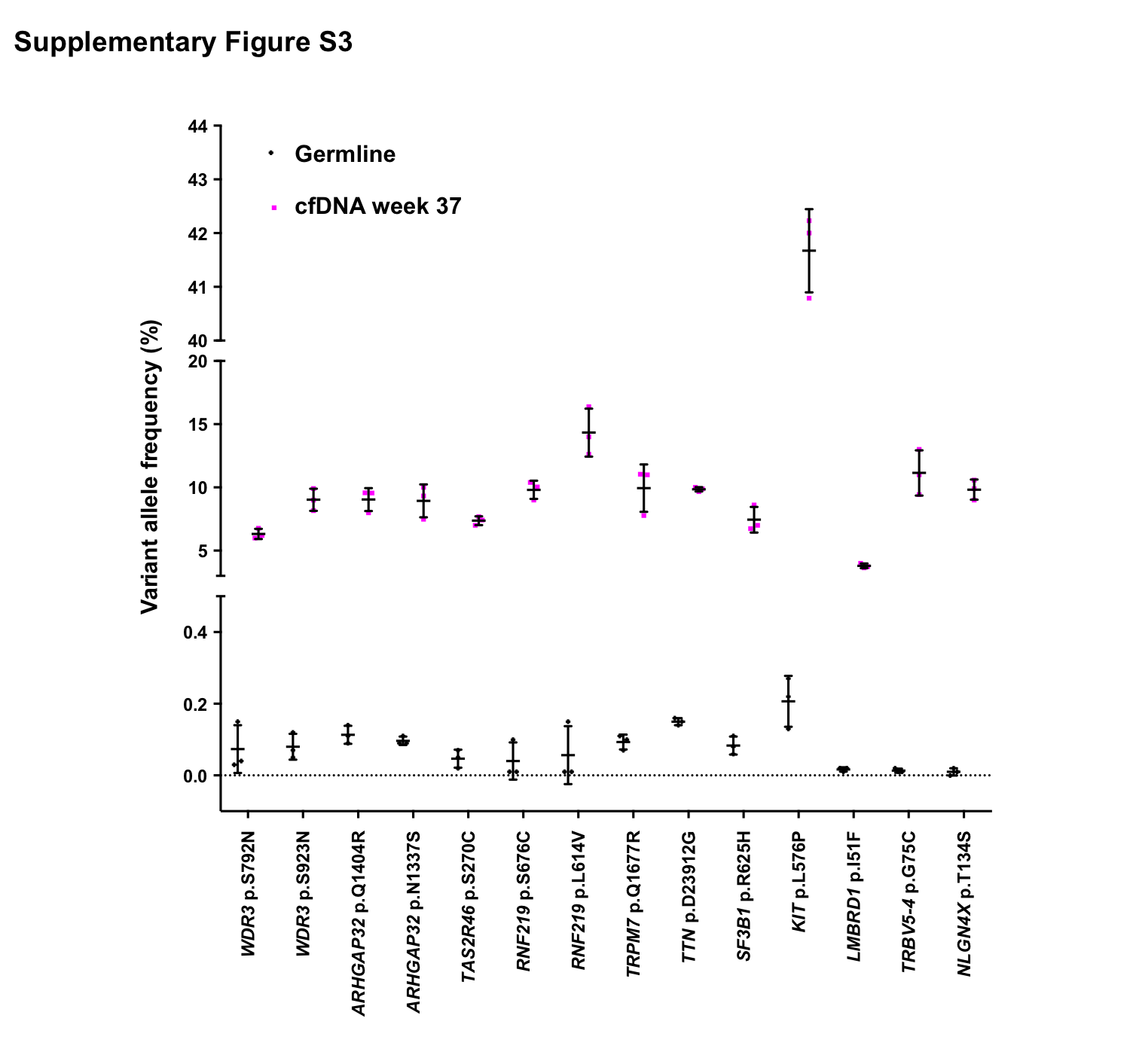

Supplement: Supplementary Data [file supp_mdw278_mdw278supp_fig3.tif]

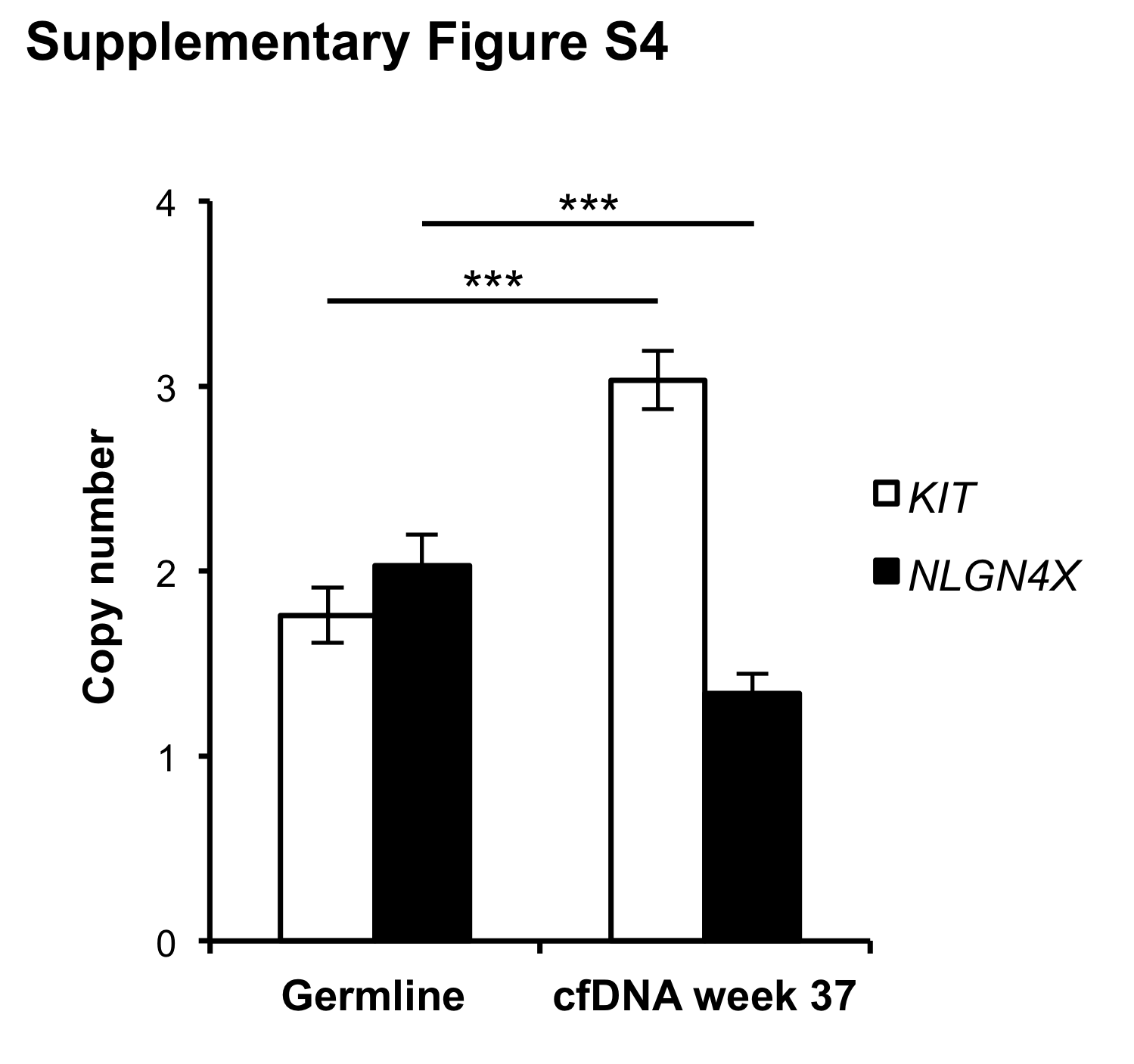

Supplement: Supplementary Data [file supp_mdw278_mdw278supp_fig4.tif]
