# Supplementary material for: Distinct subclonal tumour responses to therapy revealed by circulating cell-free DNA
Source: Ann Oncol. 2016 Aug 8;27(10):1959–65. doi: 10.1093/annonc/mdw278 (PMC5035787; doi:10.1093/annonc/mdw278)
Supplement: Supplementary Data [file supp_mdw278_mdw278supp_table1.docx]

**Supplementary Table S1.** Targeted re-sequencing amplicon characteristics.

| **Target gene** | **Amino acid change** | **Chr.** | **Chromosomal position (GRCh37)** | **Amplicon size** | **Forward primer** | **Reverse primer** |
| --- | --- | --- | --- | --- | --- | --- |
| **Single locus** | |  |  |  |  |  |
| ***KIT*** | L576P | 4 | 55593661 | 105 | TGGAAGGTTGTTGAGGAGAT | AAAACTCAGCCTGTTTCTGG |
| **Multiplex** |  |  |  |  |  |  |
| ***KIT*** | L576P | 4 | 55593661 | 103 | CAGTGGAAGGTTGTTGAGGAGA | TCAGCCTGTTTCTGGGAAACTC |
| ***TRBV5-4*** | G75C | 7 | 142168500 | 90 | TGAGAATCTAGGAGGGAAGT | ACAACACTGTGTCCTGGT |
| ***ARHGAP32*** | Q1404R | 11 | 128839808 | 101 | TATTGGGACATGACAGGCCCTT | ACCTCATGGGATCTTGAGGACA |
| ***ARHGAP32*** | N1337S | 11 | 128840009 | 89 | CAAGCTCTTTCCTTGCAGCCTA | CCTATGGCACAGTCCAGTTGAG |
| ***RNF219*** | L614V | 13 | 79190056 | 93 | GGACAAGAACTGGAATGGAGTGA | TGGAAGTGAATGGAAACCCACT |
| ***RNF219*** | S676C | 13 | 79189869 | 106 | AGGTGGTTATGAAGACTGTGCATC | CCCAGGGCATTTTGTTAAGCAG |
| ***WDR3*** | S923N | 1 | 118502006 | 82 | AAGAGGGAATGCGAGGCAAAAA | TCCTCTTCCTCTTCTTCTCTTCCA |
| ***WDR3*** | S792N | 1 | 118497216 | 95 | AGTGCTACCTAAGTCTCAGTTT | TGCCATAAGCCATTAGGATGGG |
| ***TTN*** | D23912G | 2 | 179434201 | 84 | CAAGAGAGGTGAAACTGCTA | CACCAGAAGTTAAATGGGGA |
| ***TAS2R46*** | S270C | 12 | 11214086 | 70 | CCCCAAATCAGGATGAATGGGT | CTGTCTTCATGTTCTGCGAAGC |
| ***SF3B1*** | R625H | 2 | 198267483 | 110 | GAGGCTACAACAGCAAAAGCTC | TTTTAGGCTGCTGGTCTGGCTA |
| ***TRPM7*** | Q1677R | 15 | 50866903 | 76 | GGCAAACGTAAGCTTTTGTGCT | TAAACAAGGGTTCTCCCCACCC |
| ***NLGN4X*** | T134S | X | 6069107 | 104 | TCCGTGGGCACGTAGATGTTTA | GACATGCTGCCCATCTGGTTTA |
| ***LMBRD1*** | I51F | 6 | 70500283 | 97 | CCACTGGTAGAAGTGCTGATGT | AAATACCAAAGTCGGCGGGAAA |
| ***UGT2B11**** | D458H | 4 | 70066376 | 88 | CTCGAAGGTGTTTGGCTCCTTT | CAACATGATCAACCAGTAAAGCC |

Chr., chromosome; *Amplicon did not yield specific product and was excluded from the analysis
